# Validation of the society of thoracic surgeons predicted risk of mortality score for long-term survival after cardiac surgery in Israel

**DOI:** 10.1186/s13019-022-01809-7

**Published:** 2022-04-05

**Authors:** Eyal I. Ben-David, Orit Blumenfeld, Ayelet Shapira-Daniels, Oz M. Shapira

**Affiliations:** 1 Department of Cardiothoracic Surgery, Hadassah Hebrew University Medical Center, Jerusalem, Israel; 2 The Israel Center for Disease Control, Division of Medical Technologies and Research, Ministry of Health, Ramat Gan, Israel; 3 St George’s Hospital Medical School, St George’s, University of London, Cranmer Terrace, London, SW17 0RE UK

**Keywords:** Risk prediction, Long-term survival, Logistic regression

## Abstract

**Background:**

Long-term survival is an important metric in assessing procedural value. We previously confirmed that the Society of Thoracic Surgeons predicted risk of mortality score (PROM) accurately predicts 30-day mortality in Israeli patients. The present study investigated the ability of the PROM to reliably predict long-term survival.

**Methods:**

Data on 1279 patients undergoing cardiac surgery were prospectively entered into our database and used to calculate PROM. Long-term mortality was obtained from the Israeli Social Security Database. Patients were stratified into five cohorts according to PROM (A: 0–0.99%, B: 1.0–1.99%, C: 2.0–2.99%, D: 3.0–4.99% and E: ≥ 5.0%). Kaplan–Meier estimates of survival were calculated for each cohort and compared by Wilcoxon signed-rank test. We used C-statistics to assess model discrimination. Cox regression analysis was performed to identify predictors of long-term survival.

**Results:**

Follow-up was achieved for 1256 (98%) patients over a mean period of 62 ± 28 months (median 64, range 0–107). Mean survival of the entire cohort was 95 ± 1 (95% CI 93–96) months. Higher PROM was associated with reduced survival: A—104 ± 1 (103–105) months, B—96 ± 2 (93–99) months, C—93 ± 3 (88–98) months, D—89 ± 3 (84–94) months, E—74 ± 3 (68–80) months (p < 0.0001). The Area Under the Curve was 0.76 ± 0.02 indicating excellent model discrimination. Independent predictors of long-term mortality included advanced age, lower ejection fraction, reoperation, diabetes mellitus, dialysis and PROM.

**Conclusions:**

The PROM was a reliable predictor of long-term survival in Israeli patients undergoing cardiac surgery. The PROM might be a useful metric for assessing procedural value and surgical decision-making.

## Background

In a unique collaborative, the Israeli Ministry of Health and the Israeli Society of Cardiothoracic Surgery have recently established a national adult cardiac surgery database. Approximately 4000 adult cardiac operations are performed annually in Israel [1]. The relatively small volume of cases mandates linkage to a robust database, such as that of the Society of Thoracic Surgeons (STS) [1]. The STS leadership enthusiastically endorsed the initiative, with a vision of transforming the STS National Database from national to a global quality benchmark [2].

The decision to perform any cardiac procedure is based, among many factors, on weighing the risk of the operation against the risk of alternative approaches such as percutaneous intervention and optimal medical management. Using multivariable analyses, the Society of Thoracic Surgeons 30-day Predicted Risk of Mortality score (STS PROM) was developed based upon data derived from 775,000 records out of more than 6.5 million operative records accumulated over the years in the Adult Cardiac Surgery Database (ACSDB) [3]. The models of STS PROM are updated periodically, allowing accurate risk-adjusted prediction of mortality after five cardiac surgical procedures—isolated coronary artery bypass grafting (CABG), isolated aortic valve replacement (AVR), AVR with CABG, isolated mitral valve replacement (MVR) and MVR with CABG [4]. Short-term mortality scores have been widely used for patient and procedural selection, benchmark quality metrics and means to assess cost-effectiveness [3, 4].

It has become clear to the major stakeholders in healthcare, including patients, providers, insurance agencies and other governmental bodies, that risk-adjusted long-term outcomes are essential for shared decision making, comparative studies of different treatment modalities and comprehensive evaluation of procedural value [5]. Data on the ability of short-term risk scores to predict long-term outcomes after cardiac surgery are limited [6–8].

In a large single-institution study, Puskas and colleagues demonstrated that the STS PROM was a reliable predictor of long-term survival [8]. Given the different patient characteristics, varied practice patterns and entirely different healthcare systems between Israel and the United States, the applicability of a single-US-center experience to Israel remained uncertain. Major differences between the countries in the prevalence of cardiac pathology such as rheumatic heart disease, rates of repair in mitral valve surgery, use of multiple arterial grafts for CABG, access and adherence to postoperative care, and compliance with optimal medical management, might have a profound impact on long-term survival. Therefore, the ability of a short-term risk score that has been developed in an entirely different healthcare environment must be examined.

Our institution was among the first to join the STS ACSDB [9]. We recently confirmed that the STS PROM was an accurate predictor of 30-day mortality in Israeli patients undergoing cardiac surgery [10]. In the present study we sought to investigate the ability of the STS PROM to reliably predict long-term survival after cardiac surgery in our patient population.

## Methods

### Patients

This is a retrospective cohort analysis of prospectively collected clinical data on 1279 consecutive patients who underwent five cardiac surgical procedures with a calculable STS PROM at Hadassah Hebrew University Medical Center from January of 2008 to December of 2015. The procedures included isolated coronary bypass graft grafting (CABG), aortic valve replacement (AVR) with or without CABG and mitral valve replacement or repair (MVR, r) with or without CABG.

### Operative technique

The operations were performed by a group of five surgeons via a median sternotomy, mostly (90%) using near-normothermic (34–35 °C) cardiopulmonary bypass and cardioplegic arrest. Cardiopulmonary bypass was established using ascending aortic cannula, and either a single two-stage venous cannula via the right atrial appendage, or two single-stage bi-caval cannulas for mitral valve operations. Myocardial protection was achieved using intermittent cold antegrade and retrograde blood cardioplegia. One hundred twenty-eight operations (16%) out of 798 isolated coronary artery bypass grafting were performed using standard off-pump techniques. Operative techniques remained uniform throughout the study period. Hadassah is a tertiary academic center with a training program. Two hundred eighty-one (22%) operations were performed by residents under direct supervision of a senior faculty. All faculty had gone through varied periods of post-residency advanced training in the US, partially adopting the American operative techniques and practice patterns.

### Data collection

Data were prospectively collected and entered into our STS-linked departmental database using the STS collection tool and definitions [11]. The collected data included patient demographics, risk factors, medications, extent of cardiac disease, clinical presentation, procedural details and 30-day outcomes [11]. The data were entered into the database by a specifically trained departmental database manager. To avoid bias and gaming, procedural outcomes were collected by a professional who was not affected by the results in any way. Periodical auditing against patients` medical records was routinely performed to ensure data accuracy and completeness. The STS ACSDB collection tool and risk-algorithms are updated periodically. To ensure that the calculated STS PROM for each patient in our study is accurate, we used three versions of the STS ACSDB collection tool – 2.61, 2.73 and 2.81 and the most updated risk-algorithm during the study period. The variables included in the STS PROM include the type of procedure, age, gender, height, weight, left ventricular ejection fraction, heart failure, race, renal failure, cardiac presentation, cardiac symptoms, prior myocardial infarction, cardiac arrhythmia, renal failure, peripheral vascular disease, cerebrovascular disease, peripheral arterial disease, diabetes mellitus, hypertension, immunocompromised state, endocarditis, endocarditis, coronary artery anatomy, status of surgery, cardiogenic shock, New York Heart Association functional class, intra-aortic balloon pump, inotropes, previous cardiac intervention, type and severity of aortic, mitral and tricuspid valve dysfunction and incidence of surgery (re-operation) [11]. The primary end-point of the study was all-cause mortality from the index operation to death, including perioperative deaths. Since the STS ACSDB collects 30-day mortality only, we obtained long-term survival data by linking to the National Israeli Social Security Mortality Database. We used three unique identifiers for cross-reference—the patient`s name, age and the national Identification Number that is equivalent to the social security number in the US. In Israel, the Identification Number is used as the patient`s medical record number in any medical institution throughout the patient life time.

### Statistical analysis

Categorical variables were expressed as absolute numbers and percentages. Continuous variables were expressed as means ± SD, 95% Confidence Intervals (CI), or medians with interquartile range (IQR) as appropriate. To evaluate the ability of the PROM to predict long-term survival we categorized the patients into five sub-groups according to the STS PROM—A: 0–0.99%, B: 1.0–1.99%, C: 2.0–2.99%, D: 3.0–4.99% and E: ≥ 5.0%. The calculated STS PROM was less than 5.0% in 1114 patient—89% of the cohort. To allow a meaningful comparative analysis, we based our categorization upon calculation of the median of each range of scores and the percent of patients in each score. We used the Kaplan Meier method to plot actuarial survival for each risk sub-group, and the non-parametric Wilcoxon signed-rank test to produce 95% CI and compare survival among sub-groups. To assess the discriminative power of the STS PROM risk model, we plotted the Receiver Operating Characteristics curve (ROC) and calculated the area under the curve (AUC). Model discrimination indicates the ability of the model to distinguish between survivors and non-survivors. The values of the area under the curve range from 0.5 to 1.0. Higher values indicate better model discrimination, whereas values close to 0.5 indicate that model discrimination is random. Cox regression analysis was performed to identify predictors of long-term mortality. We first performed univariable analysis of mortality using Chi square test and the Mann–Whitney test, as appropriate. Variables with a P value of ≤ 0.1 were entered into the model. The variables included in the final model were age, gender, diabetes mellitus, renal failure, dialysis, left ventricular ejection fraction, type of operation, reoperation, priority of the operation (elective, urgent, emergent, emergency salvage) and the STS PROM. We used the Spearman correlation analysis to confirm that there was no co-linearity or interactions among the covariates entered into the final model. Data were complete for most of the covariates entered into the Cox regression analysis. Missing data included: diabetes mellitus—75 patients (5.9%), left ventricular ejection fraction—22 patients (1.7%) and priority of surgery—3 patients (0.2%). A P value of < 0.05 was considered significant. The statistical analyses were performed using the SPSS version 24 for Windows (SPSS Inc., Chicago, IL, USA).

## Results

### Patients

The study cohort consisted of 1279 consecutive patients who underwent cardiac surgery at our institution between January of 2008 and December of 2015. The five surgical procedures with a calculable STS PROM that were included in the study comprised 78% of the adult cardiac operative volume in our institution during the study period. The baseline clinical and operative profiles of the study cohort are summarized in Table 1. The observed 30-day mortality was 1.9% (25 patients). The STS PROM for the entire cohort was 3.1 ± 5.9% (median 1.5%, range 0.1–96%, IQR 0.7–3.2%).

**Table 1:** Baseline clinical and surgical profiles of the study cohort

	N = 1279
Age (years)	64 ± 12 (range 20–90, median 64, IQR 56–73)
Gender (Male/female)	929 (73%) / 350 (27%)
Hypertension	818 (64%)
Diabetes mellitus	553 (43%)
Preoperative stroke	135 (11%)
Preoperative myocardial infarction	643 (50%)
Preoperative renal failure	150 (12%)
Preoperative peripheral vascular disease	158 (11%)
LVEF (%)	51 ± 12 (5–82)
Extent of coronary artery disease	
None	
Single vessel disease	286 (22%)
Double vessel disease	121 (10%)
Triple vessel disease	253 (20%)
Left main disease	619 (48%)
	268 (21%)
Operative procedure	
CABG	798 (62%)
AVR	214 (17%)
AVR ± CABG	92 (7%)
MVR, r	126 (10%)
MVR, r ± CABG	49 (4%)
Priority of surgery	
Elective	665 (52%)
Urgent	606 (47%)
Emergent or emergency-salvage	8 (1%)
Reoperation	81 (6%)

LVEF: Left ventricular ejection fraction; CABG: Coronary artery bypass grafting; AVR: Aortic valve replacement; MVR, r: Mitral valve replacement or repair

### Long-term survival

**Fig. 1:**
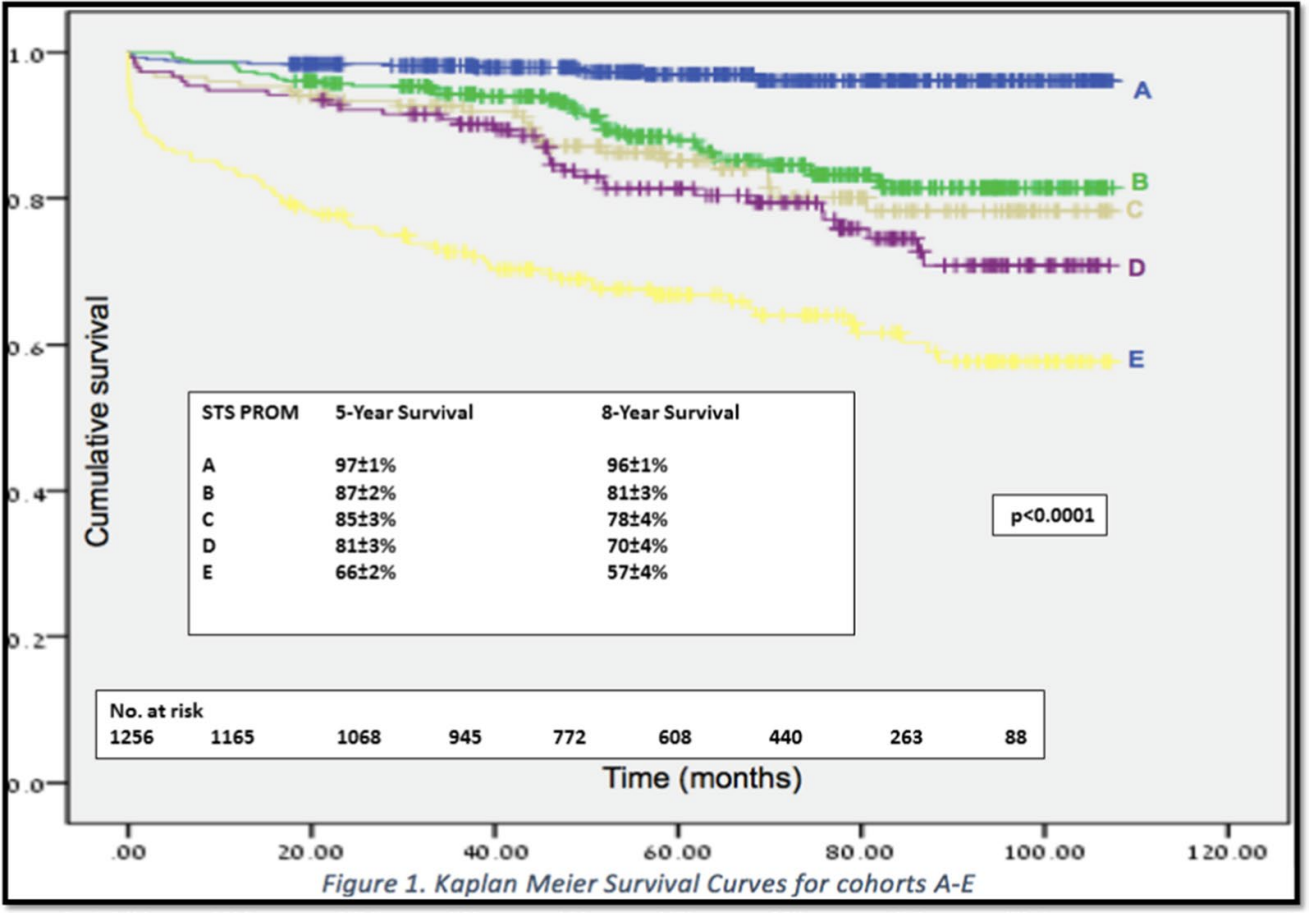
Kaplan–Meier survival estimates for cohorts A-E. Kaplan–Meier survival estimates stratified by the Society of Thoracic Surgeons Predicted Risk of Mortality. The cohort was divided into five sub-groups according to the Society of Thoracic Surgeons Predicted Risk of Mortality—A: 0–0.99%, B: 1.0–1.99%, C: 2.0–2.99%, D: 3.0–4.99% and E: ≥ 5.0%. Higher Society of Thoracic Surgeons Predicted Risk of Mortality was a strong predictor of decreased long-term survival. P < 0.0001 by the non-parametric Wilcoxon signed-rank test of each risk category against Group A

Complete follow-up was achieved for 1256 (98%) patients over a mean period of 62 ± 28 months (range 0–107 months, median 64, IQR 41–86). Mean survival of the entire cohort was 95 ± 1 months (95% CI 93–96). Higher STS PROM was associated with reduced survival: A—104 ± 1 (CI 103–106) months, B—96 ± 2 (CI 93–99) months, C—93 ± 3 (CI 88–98) months, D—89 ± 3 (CI 84–95) months, E—74 ± 3 (CI 68–81) months (Table 2, Fig. 1). Incremental increase in the STS PROM was associated with a significant decrease in survival (Wilcoxon signed-rank test, p < 0.0001 comparing each subgroup against A or E). Survival among groups B, C and D was similar with overlapping confidence intervals.

**Table 2:** Patient survival stratified according to the STS PROM

Risk subgroup (n)	STS PROM (%)	Mean ± SEM survival (months)	95% confidence intervals	8-Year Survival (%)
A (457)	0–0.99	104 ± 1	103–106	96 ± 1
B (306)	1.0–1.99	96 ± 2	9 3–99	81 ± 3
C (151)	2.0–2.99	93 ± 3	88–98	78 ± 4
D (153)	3.0–4.99	89 ± 3	84–95	70 ± 5
E (189)	> 5.0	74 ± 3	68–81	57 ± 4
Total (1279)	3.1	95 ± 1	93–96	81 ± 1

STS PROM: Society of Thoracic Surgeons Predicted Risk of Mortality; SEM: Standard error of the mean

### Model discrimination

**Fig. 2:**
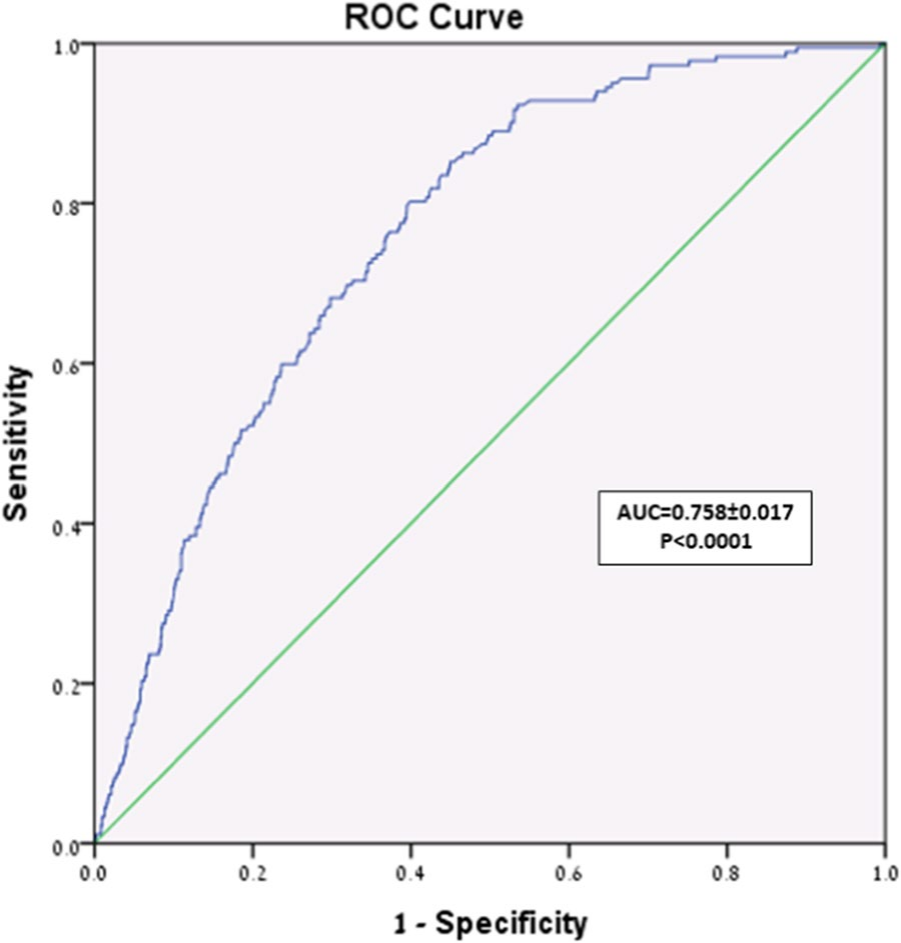
Area under Receiver Operator Curve. Receiver Operating Characteristics Curve of the Society of Thoracic Surgeons Predicted Risk of Mortality model. Model discrimination indicates the ability of the model to distinguish between survivors and non-survivors. The values of the area under the curve range from 0.5 to 1.0. Higher values indicate better model discrimination, whereas values close to 0.5 indicate that the model discrimination is random. The Area Under the Curve was 0.76 ± 0.02, indicating excellent model discrimination (p < 0.0001, Wilcoxon signed-rank test)

To assess model discrimination of the STS PROM for overall survival we plotted the ROC and measured AUC (Fig. 2). The AUC for the STS PROM model was 0.76 ± 0.02 indicating excellent model discrimination for overall survival.

### Predictors of mortality

In a cox regression analysis, advanced age, lower ejection fraction, reoperation, diabetes mellitus, preoperative dialysis and STS PROM were identified as independent predictors of long-term mortality (Table 3). Higher STS PROM was associated with a significant increase in the hazard ratio for long-term mortality.

**Table 3:** Independent predictors of long-term mortality

Risk factor	Hazard ratio	95% confidence intervals	P value
Age > 65 years	1.6	1.1–2.3	0.02
Diabetes mellitus	1.5	1.1–2.0	0.02
Preoperative dialysis	2.5	1.2–4.9	0.01
Reduced LVEF	1.6	1.1–2.5	0.005
Reoperation	2.0	1.2–3.2	0.005
STS PROM*			
B C D E	3.3 3.4 4.3 6.3	1.7–6.3 1.7–7.4 2.2–8.7 3.1–12.6	< 0.001 < 0.001 < 0.001 < 0.001

STS PROM—Society of Thoracic Surgeons Predicted Risk of Mortality; LVEF—Left ventricular ejection fraction; *—Hazard ratio compared to group A (STS PROM = 0—0.99%)

## Discussion

Long-term outcomes have become essential components in the assessment of procedural value [5]. In fact, it is imperative that long-term survival data after surgical, catheter-based interventions and conservative (non-interventional) treatments will be available to patients and healthcare providers for surgical decision-making [12]. Obtaining long-term outcomes is also a fundamental requirement in the transition from a procedural-specific to a disease-specific cardiovascular database (e.g. coronary artery or aortic valve disease), using a model recently developed in the Netherlands [13].

The determinants of long-term outcomes after cardiac surgery include preoperative patient characteristics, extent of cardiac disease, procedural details, early postoperative complications, and access and adherence to postoperative care. Many of these determinants are used to generate early post-cardiac surgery risk scores such as the STS PROM. In a previous study, we evaluated the STS PROM, Logistic EuroSCORE I and EuroSCORE II, and showed that the STS PROM was a reliable metric for 30-day mortality in Israeli patients undergoing cardiac surgery [10]. Due to logistical, administrative, political and financial barriers, most large-scale clinical registries, including the STS ACSDB, collect only 30-day outcomes. To overcome this limitation and ascertain that the data collected on study primary end-point of all-cause mortality are complete and accurate, we adopted a methodology used by Weintraub and colleagues in the ASCERT trial and linked our clinical database to the National Israeli Social Security Mortality Database—a very reliable administrative database [14]. In the present study, we demonstrated that the STS PROM was also a reliable predictor long-term survival in our patient population. Model calibration and model discrimination were excellent as evidenced by a significant decrease in patient survival with each increment in the STS PROM, and a high value of the Area Under the Receiver Operating Characteristics, respectively.

The findings in our study are in line with a study of Puskas and colleagues who showed that the STS PROM was a reliable predictor of long-term mortality in 24,222 patients who underwent the five cardiac operations with a calculable PROM in their institution [8]. They observed excellent model calibration and discrimination at 1, 3, 5 and 10 years after surgery [8].

The fact that the STS PROM was a reliable predictor of long-term survival in two different healthcare environments is quite remarkable and somewhat unexpected. There are substantial differences between Israel and the US in factors that might have a profound effect on long-term outcomes. These include differences in patient characteristics, varied practice patterns, variability in access to care and adherence to follow up.

For example, there is fundamental difference between US and Israel in regards to race. Between 9.5 to 17.2% of patients included in the study of Puskas and colleagues were African-Americans compared with none in our study. African-American race is an important variable in the STS risk algorithm [3]. African-American race is associated with lower socio-economic status, limited access to care of worse cardiovascular health [15]. Similarly, the percentage of Hispanic, Latino, Spanish, or Asian patients in Israel is negligible. In contrast, these ethnical groups, which are associated with worse outcomes compared to whites, comprise a significant and increasing proportion of patients undergoing cardiac surgery in the US.

Cardiac pathology is also different between the countries. For example, the prevalence of rheumatic heart disease is higher in Israel compared to the US [16]. The repair rate and the durability of repair of rheumatic mitral valve pathologies are lower compared to myxomatous disease—the most common valve pathology in the US.

Two striking differences between US and Israel relate to patients undergoing CABG. During the past two decades, there was a dramatic shift of patients requiring coronary revascularization from CABG to percutaneous revascularization in Israel [1]. In fact, the ratio of percutaneous to surgical revascularization of 8.5:1 in Israel is among the highest in the world and is substantially higher than the US and the Organization for Economic Cooperation and Development (OECD) [1]. Thus, it is possible that patients referred for CABG in Israel are at higher risk compared to the OECD. Another factor relates to graft selection. Despite growing body of evidence, the use of multiple arterial grafts for CABG remains low in the US [17]. In a recent report on nearly 1.5 million CABG operations, the rates of bilateral internal mammary artery and radial artery grafting strategy utilization were 4.9% and 6.5%, respectively [17]. In contrast, in our study arterial grafts utilization rates were substantially higher—25% and 50%, respectively.

Another important difference between US and Israel that may have an impact on long-term outcomes after cardiac surgery relates to variability in access to care. One third of our patients were Palestinians living in the west bank, Gaza, or east Jerusalem. Many of them had limited access to high quality postoperative care, adversely affecting their long-term survival.

The ability of the STS PROM to predict long-term mortality so precisely was also quite unexpected given that the STS ACSDB collection tool did not include medical variables such as preoperative chest irradiation and liver disease, any type of frailty index and socioeconomic data such as level of education and household income. This issue, affecting both studies, is one of the limitations of the STS PROM. We plan to expand our institutional database and systematically collect these data to strengthen the prediction model.

Despite these differences, the remarkable similarity between the study by Puskas and colleagues and ours attests to the strength of the STS PROM related to the robust dataset used to develop the model and its periodical updates. These findings also suggest that once validated for short-term outcomes, the STS PROM is likely to be a reliable predictor of long-term survival in the tested population.

This study has important limitations. The study cohort was relatively small and heterogeneous. The small number of patients precluded sub-group analyses with respect to each of the five cardiac surgical procedures. Due to the small cohort, we categorized the patients into five similar-size risk subgroups for the purpose of model calibration. We used similar methodology to that used by Pulkas and colleagues who divided their cohort into ten deciles based on the STS PROM [8]. This methodology is different compared to the more clinically-oriented categorization into three groups—low risk (PROM ≤ 3%), medium risk (3–8%) and high risk (> 8%) that is commonly used in patients with aortic stenosis [18]. Performing multicenter trial enrolling a large number of patients, using the recently established Israel STS-linked ACSDB [2], is clearly indicated to overcome these limitations and confirm our findings on a national-level. Finally, we were unable to collect the cause of death. Distinguishing from cardiac-related to non-cardiac related deaths would have enhanced our study.

## Conclusions

Despite the aforementioned limitations, we conclude that the 30-day STS-PROM was a strong and reliable predictor of long-term survival in Israeli patients undergoing cardiac surgery. The long-term survival implications derived from the STS PROM might be very useful in assessing procedural value and surgical decision-making.

## Data Availability

The datasets used and/or analysed during the current study are available from the corresponding author on reasonable request.

## Abbreviations

STS: Society of Thoracic Surgeons
PROM: Predicted Risk of Mortality
ACSDB: Adult Cardiac Surgery Database
CABG: Coronary Artery Bypass Graft
MVR: Mitral Valve Replacement
MVr: Mitral Valve Repair
AVR: Aortic Valve Replacement
ROC: Receiver Operating Characteristics curve
AUC: Area under the curve
OECD: Organisation for Economic Cooperation and Development

## References

[1] Blumenfeld O, Na’amnih W, Shapira-Daniels A, Lotan C, Shohat T, Shapira OM (2017). Trends in coronary revascularization and ischemic heart disease-related mortality in Israel. J Am Heart Assoc.

[2] Shapira OM, Blumenfeld O, Bolotin G, Grover FL, Shahian DM (2017). International participation in the Society of Thoracic Surgeons Adult Cardiac Surgery Database—From Institutional to National. Ann Thorac Surg.

[3] Dagostino RS, Jacobs JP, Badhwar V (2016). The Society of Thoracic Surgeons Adult Cardiac Surgery database: 2016 update on outcomes and quality. Ann Thorac Surg.

[4] Shahian DM (2016). The Society of Thoracic Surgeons National Database: “What`s past is prologue”. Ann Thorac Surg.

[5] Porter EM (2010). What is value in health care?. N Eng J Med.

[6] Heikkinen J, Biancari F, Satta J (2007). Predicting immediate and late outcome after surgery for mitral valve regurgitation with EuroSCORE. J Heart Valve Dis.

[7] Kobayashi KJ, Williams JA, Nwakanma LE (2009). EuroSCORE predicts short- and long-term mortality in combined aortic valve replacement and coronary artery bypass patients. J Cardiac Surg.

[8] Puskas JD, Kiglo PD, Thourani VH (2012). The Society of Thoracic Surgeons 30-day predicted risk of mortality score also predicts long-term survival. Ann Thorac Surg.

[9] Shapira OM, Badhwar V, Shahian D (2014). International participation in the Society of Thoracic Surgeons National Database—Mapping the journey from dream to reality. Ann Thorac Surg.

[10] Shapira-Daniels A, Blumenfeld O, Carranza A, Korach A, Rudis E, Izhar U, Shapira OM**.** Comparison of the Society of Thoracic Surgeons predicted risk of mortality, Logistic EuroScore I and EuroScore II in Israeli patients undergoing cardiac surgery. 2017 STS/EACTS Latin America Cardiovascular Surgery Conference. Cartagena, Columbia.

[11] http://www.sts.org/sts-national-database/database-managers/adult-cardiac-surgery-database/data-collection#data. Accessed 24 Jan 2017.

[12] Noorani A, Hippelainen M, Nashef SAM (2014). Time until treatment equipoise. A new concept in surgical decision making. JAMA Surg.

[13] Van Veghel D, Marteijn M (2016). de Mol B on behalf of the Measurably Better Study Group (The Netherlands) and Advisary Board. Eur J Carrdiothorac Surg.

[14] Weintraub WS, Grau-Sepulveda MV, Weiss JM, et al. Comparative effectiveness of revascularization strategies. N Engl J Med 2012;1467–76. 10.1056/NEJMoa1110717 PMC4671393 22452338

[15] Pool LR, Ning H, Lloyed-Jones DM, Allen NB (2017). Trends in racial/ethnic disparities in cardiovascular health among US adults from 1999–2012. J Am Heart Assoc.

[16] Seckeler MD, Hoke TR (2011). The worldwide epidemiology of rheumatic fever and rheumatic heart disease. Clin Epidemiol.

[17] Schwann TA, Habib RH, Wallace E, Shahian DM, O’Brien S, Jacobs JP (2018). Operative outcomes of multiple-arterial versus single-arterial coronary bypass grafting. Ann Thorac Surg.

[18] Reardon MJ, Van Mieghem NM, Pompa JJ (2017). Surgical or transcatheter aortic-valve replacement in intermediate-risk patients. N Engl J Med.

